# *Cryptosporidium* infections in asymptomatic calves up to 4 months in Poland: a cross-sectional population study

**DOI:** 10.1038/s41598-023-47810-5

**Published:** 2023-11-28

**Authors:** Artur Rzeżutka, Agnieszka Kaupke

**Affiliations:** Department of Food and Environmental Virology, National Veterinary Research Institute, Al. Partyzantów 57, 24-100 Puławy, Poland

**Keywords:** Molecular biology, Parasitology

## Abstract

Cattle cryptosporidiosis is noted worldwide with varied frequency of infection prevalence depending on geographical, environmental and husbandry factors. In this study, the prevalence of *Cryptosporidium* infections in cattle was determined on the basis of molecular results obtained by testing 1601 faecal samples collected from calves up to 4 months of age housed in all Polish provinces from 2014 to 2018. Detection and identification of *Cryptosporidium* species was performed at the 18 small subunit ribosomal RNA (*18S rRNA*) locus by conducting PCR–RFLP analysis of the amplified DNA fragments. The prevalence of *Cryptosporidium* infections in the cattle population was 45.3% (CI 95%: 42.8–47.7; 725/1601). The infected animals were housed on 233/267 (87.3%) of monitored farms with regional prevalence ranging from 27.8 to 62%. The restriction pattern of *18S rRNA* amplicons for positive samples was characteristic of *C*. *parvum*, *C. bovis*, *C. ryanae*, *C. andersoni*, and unexpectedly also of *C. baileyi* and *C. suis*. Infections of *C. bovis* and *C. ryanae* prevailed in the studied cattle population relegating *C. parvum* to third in prevalence. Likewise, mixed infections caused by *C. bovis* and *C. ryanae* as well as *C. parvum* and *C. bovis* were observed. A relationship between the infecting parasite species and animal breed was found. For instance, *C. parvum* prevailed in Black and White lowland breed, *C. ryanae* in Limousine cattle and *C. andersoni* in dairy animals of mixed dairy breeds. Furthermore, differences in prevalence of particular parasite species between cattle breeds were also shown.

## Introduction

*Cryptosporidium* infections in cattle were recognised for the first time in 1971 in the United States of America in an 8-month-old diarrhoeic female calf. Histopathology of the small intestine revealed atrophy of the villi and the presence of various developmental forms of *Cryptosporidium* in the epithelium^1^. Since then, *Cryptosporidium* infections and cryptosporidiosis have been reported in cattle worldwide^2^. The significant role of cattle as a source of *Cryptosporidium* for humans was recognised during a waterborne epidemic of human cryptosporidiosis in Milwaukee, USA^3^. Likewise in Poland, studies on occurrences of *Cryptosporidium* in bovine host have been conducted since the 1970s, usually being confined to animals bred on farms from selected Polish regions^4–6^ and using methods not always conducive to identification of the parasite species^4,7,8^. However, an attempt to assess the prevalence of infections nationwide by individual *Cryptosporidium* species in Polish cattle of different ages has also been undertaken^9^. As a pilot study, it covered a broad group of animals at the age from 1 day to 6 years of different health status. However, due to the low number of samples obtained from particular Polish provinces, the results did not allow for a regional assessment of the infection prevalence as well as to gather unbiased data on the occurrence of *Cryptosporidium* species in the population. It has been shown that infections caused by different *Cryptosporidium* species mainly affect animals up to the age of 4 months with *Cryptosporidium bovis* (*C. bovis*) as predominating species.

A wider application of molecular tools in veterinary parasitology has facilitated identification of *Cryptosporidium* species in livestock. As with other similar studies conducted in Europe, in Poland the infections previously diagnosed in cattle were mostly caused by *Cryptosporidium parvum* (*C. parvum*)^8,10,11^. The tested animals were also positive for *C. bovis*, *Cryptosporidium andersoni* (*C. andersoni*) and *Cryptosporidium ryanae* (*C. ryanae*)^9,11^. An age-related pattern of *Cryptosporidium* species infecting cattle was solely observed for *C. parvum*. *C. andersoni* was the only species occurring in adult cattle over one year of age^9^. Species which were unusual for the bovine host, such as *Cryptosporidium felis* (*C. felis*), were only occasionally detected in cattle populations in Poland^12^.

In an effort to gather data on the prevalence of *Cryptosporidium* infections in cattle population and in particular Polish provinces, only healthy animals up to the age of 4 months were sampled. Apart from a molecular assessment of the infection prevalence, the study aimed to identify parasite species and their regional distribution pattern in cattle of different breeds and ages.

## Materials and methods

### Ethics approval and consent to participate

This study did not require approval of an Ethics Committee. However, freshly voided faeces were collected during routine veterinary practice in adherence to international guidelines for animal care. Sample collection was not harmful and did not violate animal welfare laws. No clinical interventions were performed. The owners of the cattle included in this study provided informed consent to participate.

### Cattle faeces

Over a period of 5 years, 1601 freshly voided faecal samples were collected from cattle from the age of 1 week to 4 months from 2014 to 2018 (Table 1). Each cattle was sampled only once and the sampling plan covered each year different provinces. The animals were housed on 267 farms located across the 16 administrative provinces of Poland. The average number of animals in the herd was 137. The cattle were mainly raised on large farms (175, 65.5%) with > 50 heads. Small farms (< 50 heads) accounted for 92 (34.5%) of the tested establishments. In each year from 306 to 324 faeces were subjected for testing. Animals originated from farms located in districts with the highest cattle populations in 3 or 4 provinces each year^13^. In each province 18 farms were monitored except from Dolnośląskie (DS) (15 farms) and Opolskie (OP), Śląskie (SL) and Podkarpackie (PK) where 12 farms were sampled in each localization. Farms were randomly selected and represented different administrative locations in the province. Generally, from each farm freshly voided faeces of 6 animals (18 per district) of different breeds at the age between 1 week to 4 months were taken. However, there were also farms enrolled on which all sampled animals were the same age and breed. For instance, on 9 farms, there were only 4-week-old calves and on 15 farms (5 with calves of each age) there was stock at the age of 8, 12, and 16 weeks. The cattle were in good health without symptoms of *Cryptosporidium* infections. During this study no clinical intervention or animal examination were conducted by vets taking care of these farms who collected samples. The animals were divided into three age groups as shown in Table 1. Faecal samples of 10–15 g were placed individually into plastic containers, labelled and sent to the laboratory. Faeces were collected from cattle representing dairy breeds (Polish Black and White Holstein*–*Friesian (HO), Jersey (JE), Polish Red and White Holstein*–*Friesian (RW), Brown Swiss (BS), and dairy animals of mixed dairy breeds (MS)); meat breads (meat cattle of mixed meat breeds (MM), Aberdeen Angus (AN), Charolaise (CH), Salers (SL), Limousine (LM), and Belgian Blue (BB)); and cattle of mixed dairy-meat breeds (mixed dairy-meat breeds (MDM), Black and White lowland (NCB), Polish Red (RP), Montbéliarde (MO), Polish Black and White (ZB) and Simmental (SM)). The sampled animals mainly represented dairy breeds (74.8%), followed by meat (14.5%) and mixed (10.7%) breeds.

**Table 1:** However, greater than 40% prevalence of infections was revealed in the majority of the monitored regions. The number of faecal samples collected from cattle up to the age of four months housed in different Polish provinces.

Province	Number of animals in age groups (weeks)			Total
	≥ 1–4	> 4–8	> 8–16	
Dolnośląskie (DS)	14	32	44	90
Kujawsko-Pomorskie (KP)	27	34	47	108
Lubelskie (LB)	46	41	21	108
Lubuskie (LS)	58	22	28	108
Łódzkie (LD)	33	25	50	108
Małopolskie (MP)	18	26	64	108
Mazowieckie (MZ)	46	29	33	108
Opolskie (OP)	8	22	42	72
Podkarpackie (PK)	5	31	36	72
Podlaskie (PL)	23	39	46	108
Pomorskie (PM)	78	8	22	108
Śląskie (SL)	4	30	38	72
Świętokrzyskie (SK)	43	45	20	108
Warmińsko-Mazurskie (WM)	36	30	42	108
Wielkopolskie (WP)	35	21	51	107
Zachodniopomorskie (ZP)	53	29	26	108
Total	527	464	610	1601

### Detection and identification of *Cryptosporidium* species based on the analysis of the *18S rRNA* gene fragment

*Cryptosporidium* DNA was extracted from 0.1 g (100 µl) of the faeces with an alkali wash and a heat lysis method developed by Millar et al.^14^ with further modifications^9^. Nucleic acid extracts were purified using a GeneMATRIX PCR/DNA Clean-Up Purification Kit (EURx Ltd., Gdańsk, Poland) according to the manufacturer’s instructions. The extracts containing parasite DNA were stored at − 20 °C until use. Detection of *Cryptosporidium* DNA was performed by a nested-PCR method allowing amplification of the *18S rRNA Cryptosporidium* gene fragment using the primers specified by Xiao et al.^15^ and conditions described elsewhere^16^. To increase amplification efficiency and to reduce amplification inhibition each reaction mixture was supplemented with 20 µg of bovine serum albumin (Thermo Fisher Scientific, Vilnius, Lithuania)^9^. The correct performance of the nucleic acid extraction and the amplification step were verified by inclusion of an appropriate set of controls as described elsewhere^9^. All reactions were performed in a Biometra TProfessional BASIC thermocycler (Analytik Jena, Jena, Germany).

The identity of *Cryptosporidium* species in positive faeces was defined by a PCR–RFLP analysis of the amplified *18S rRNA* fragments. The analysis used the following enzymes: *Nde*I for identification of *C. parvum*^17^, *Mbo*II in a parallel digestion for *C. bovis* and *Cryptosporidium ryanae* (*C. ryanae*) detection^18^, *Xba*I for differentiation of *C. andersoni* and *C. parvum* by their lack of a restriction site from *C. bovis* and *C. ryanae*^9^, *Bcu*I (*Spe*I) in digestion for identification of *Cryptosporidium suis* (*C. suis*)^17^ and *Ssp*I for detection of *Cryptosporidium baileyi* (*C. baileyi*)^19^. To confirm the correct identification of *Cryptosporidium* species which were unusual for cattle such as *C. suis* and *C. baileyi*, sequencing of the post-PCR *18S rRNA* amplicons was conducted. The PCR products excised from the agarose gel and purified were directly sequenced in both directions using the ABI Prism BigDye Terminator v3.1 Cycle Sequencing Kit on an ABI 3730XL DNA sequencer (Life Technologies, Carlsbad, CA, USA) at the Genomed S.A. sequencing service (Warsaw, Poland). Nucleotide sequences were aligned with published sequences from GenBank by using the NCBI-BLAST program (http://blast.ncbi.nlm.nih.gov/Blast.cgi). The *18S rRNA* nucleotide sequences of *C. baileyi* and *C. suis* were deposited in GenBank under the accession numbers OP090504-OP090507.

### Statistical analyses

The prevalence of *Cryptosporidium* infections in cattle in particular Polish provinces was estimated by the Clopper-Pearson method. It was also used to assess prevalence of infections in age groups of animals as well as age-related prevalence of detected parasite species. Subsequently, a chi-squared (χ2) test with Yates' continuity correction was employed to assess infection prevalence between age groups of animals and to determine dominating *Cryptosporidium* species in cattle. Concluding the statistical work, the uncorrected χ2 test and odds ratio in logistic regression was employed to analyse differences in regional infection prevalence in animals from different age groups, prevalence of *Cryptosporidium* species between provinces and animal breeds. The calculations were performed using R software v. 4.1.1^20^ with "prevalence"^21^ and “epiR”^22^ packages.

## Results

### Detection of *Cryptosporidium* infections in cattle

The *18S rRNA* gene fragment was successfully amplified in 725 out of 1601 cattle faecal samples (Table 2). The restriction pattern of *18S rRNA* amplicons characteristic for *C. parvum*, *C. bovis*, *C. ryanae*, and *C. andersoni* was shown for 100, 368, 211, and 54 DNA samples respectively. Digestion by *SSp*I revealed the presence of *C. baileyi* in two samples. Two samples were also positive for *C. suis* when treated with *SSp*I and *Bcu*I (*Spe*I). Twelve samples contained mixtures of two different sequences such as *C. parvum* and *C. bovis* and *C. bovis* and *C. ryanae*. Sequence analysis of the *18S rRNA* fragments of *C. baileyi* and *C. suis* confirmed the species identity.

**Table 2:** The number of *Cryptosporidium* positive faeces collected from cattle up to the age of four months housed in different Polish provinces.

Province (number of tested animals)	Number of farms		Number/percentage of positive animals in age groups (weeks)			Total (%)
	Tested	Positive	≥ 1–4 (n = 527)	> 4–8 (n = 464)	> 8–16 (n = 610)	
PL (n = 108)	18	17	12 (11.1)	25 (23.1)	28 (25.9)	67 (62.0)
ZP (n = 108)	18	18	22 (20.4)	14 (13.0)	24 (22.2)	60 (55.6)
SK (n = 108)	18	16	24 (22.2)	22 (20.4)	11(10.2)	57 (52.8)
PM (n = 108)	18	17	41 (38.0)	3 (2.8)	10 (9.2)	54 (50.0)
KP (n = 108)	18	16	17 (15.7)	14 (13.0)	20 (18.5)	51 (47.2)
DS (n = 90)	15	15	3 (3.33)	19 (21.1)	26 (28.9)	48 (53.3)
MZ (n = 108)	18	14	24 (22.2)	14 (13.0)	10 (9.3)	48 (44.4)
LS (n = 108)	18	13	28 (25.9)	7 (6.5)	10 (9.3)	45 (41.7)
LB (n = 108)	18	17	16 (14.8)	21 (19.4)	7 (6.5)	44 (40.7)
LD (n = 108)	18	15	15 (13.9)	9 (8.3)	19 (17.6)	43 (39.8)
WP (n = 107)	18	17	14 (13.0)	8 (7.5)	21 (19.6)	43 (40.2)
WM (n = 108)	18	15	12 (11.1)	13 (12.0)	11 (10.2)	36 (33.3)
OP (n = 72)	12	10	5 (6.9)	14 (19.4)	16 (22.2)	35 (53.3)
SL (n = 72)	12	11	2 (2.8)	17 (23.6)	14 (19.4)	33 (45.8)
PK (n = 72)	12	10	2 (2.8)	18 (25.0)	11 (15.3)	31 (43.0)
MP (n = 108)	18	12	2 (1.8)	9 (9.3)	19 (17.6)	30 (27.8)
Total	267	233	239	227	257	725

DS—Dolnośląskie, KP—Kujawsko-Pomorskie, LB—Lubelskie, LD—Łódzkie, LS—Lubuskie, MP—Małopolskie, MZ—Mazowieckie, OP—Opolskie, PK—Podkarpackie, PL—Podlaskie, PM—Pomorskie, SK—Świętokrzyskie, SL—Śląskie, WM—Warmińsko-Mazurskie, WP—Wielkopolskie, ZP—Zachodniopomorskie.

### Geographical distribution of* Cryptosporidium* infections

**Figure 1:**
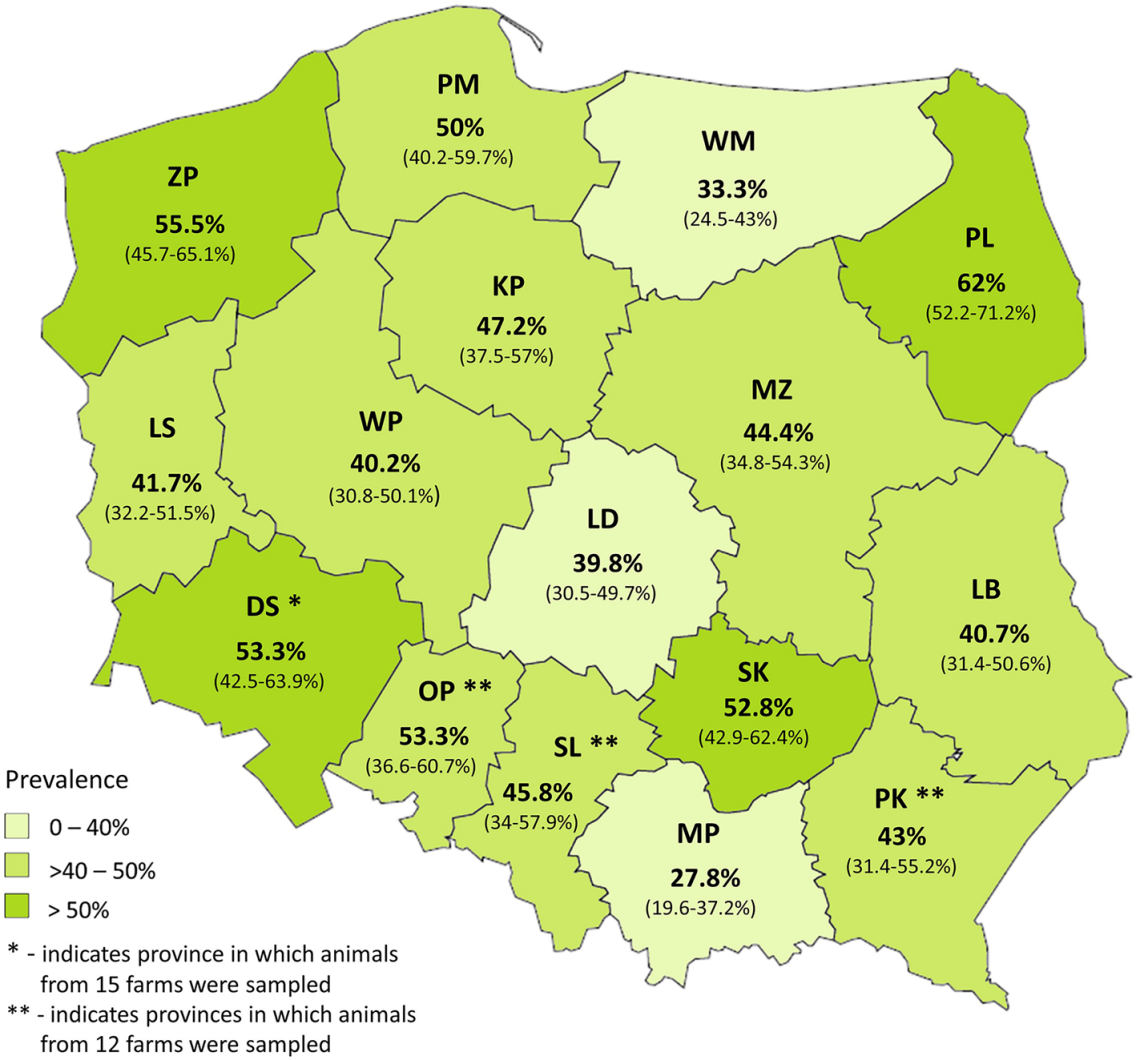
Province-related prevalence of *Cryptosporidium* infections in cattle. The numbers within parentheses indicate 95% CI.

The infected animals were reared on 233 (87.3%) out of 267 monitored farms located across all 16 Polish provinces. In the cases of the Zachodniopomorskie (ZP) and DS provinces, all monitored farms appeared *Cryptosporidium* positive. *Cryptosporidium* infections were detected with varied prevalences ranging from 27.8% in Małopolskie (MP) to 62% in Podlaskie (PL) province (Fig. 1). However, greater than 40% prevalence of infections was revealed in the majority of the monitored regions﻿.﻿

A relationship between the prevalence of *Cryptosporidium* infections and the age groups of cattle farmed in particular provinces was shown. In calves at 1–4 weeks of age, the highest number of infections was detected in Kujawsko-Pomorskie (KP) and Świętokrzyskie (SK) compared to the following provinces: DS (χ^2^ = 6.4; *p* = 0.012; OR 6.2 and χ^2^ = 5.0; *p* = 0.025; OR 4.6), Lubelskie (LB) (χ^2^ = 5.4; *p* = 0.02; OR 3.2 and χ^2^ = 4.0; *p* = 0.046; OR 2.4), Warmińsko-Mazurskie (WM) (χ^2^ = 5,4; *p* = 0.02; OR 3.4 and χ^2^ = 4,0; *p* = 0.046; OR 2.5) and MP (χ^2^ = 11.9; *p* < 0.001; OR 13.6 and χ^2^ = 10.4; *p* = 0.001; OR 10.1). Significant differences in prevalence of infections were also seen between DS and Mazowieckie (MZ) (χ^2^ = 4.1; *p* = 0.043; OR 0.25) and between all provinces except DS, LB, WM, SL, and PK relative to MP (χ^2^ = from 11.9 to 4.7; p ≤ 0.03; OR from 13.6 to 5.3). High percentages of infected animals at the age of > 4–8 weeks were found in PL compared to KP (χ^2^ = 5.8; *p* = 0.016; OR 3.21), LS (χ^2^ = 8.0; *p* = 0.005; OR 4.82), Łódzkie (LD) (χ^2^ = 6.8; *p* = 0.009; OR 4.0), Wielkopolskie (WP) (χ^2^ = 5.4; *p* = 0.02; OR 3.7), and MP (χ^2^ = 7.6; *p* = 0.006; OR = 4.2). They were also observed between DS and Lubuskie (LS) (χ^2^ = 4.0; *p* = 0.046; OR 3.1), OP and MP (χ^2^ = 4.0; *p* = 0.045; OR 3.3), LS and WM (χ^2^ = 3.9; *p* = 0.048; OR 0.3), LS and OP (χ^2^ = 4.5; *p* = 0.035; OR 0.3). Generally, *Cryptosporidium* infections in the oldest group of calves predominated in PL (χ^2^ = 6.1; *p* = 0.013; OR 0.5) comparing to other provinces except for SK, Pomorskie (PM), KP, and DS. Likewise, animals at that age were more frequently infected in ZP than in other Polish provinces (χ^2^ = 25.4; *p* < 0.001; OR 0.06). Significant differences were also found between the following provinces DS and MZ (χ^2^ = 6.3; *p* = 0.012; OR 3.3 ), LD (χ^2^ = 4.2; *p* = 0.041; OR 2.4), WM (χ^2^ = 14; *p* < 0.001; OR 5.2), PK (χ^2^ = 6.5; *p* = 0.011; OR 3.3), and MP (χ^2^ = 9.3; *p* = 0.002; OR 3.4) as well as between WP and WM (χ^2^ = 4.5; *p* = 0.033; OR 2.5), WM and SL (χ^2^ = 6.7; *p* = 0.009; OR 0.28).

### Age related prevalence of *Cryptosporidium* spp*.*

Overall *Cryptosporidium* infection prevalence for the studied cattle population was 45.3% (CI 95%: 42.8–47.7). Parasites were detected in all age groups of animals with the following group prevalence: 45.3% (CI 95%: 41–49.7; 1–4 weeks), 48.9% (CI 95%: 44.3–53.6; 4–8 weeks), and 42.4% (CI 95%: 38.5–46.5; 8–16 weeks) (Table 3).

**Table 3:** Results on detected *Cryptosporidium* species in cattle in Poland.

*Cryptosporidium* species	Number / percentage of positive animals Age of animals (weeks)				Number / infection percentage
	≥ 1–4 (n = 527)	> 4–8 (n = 464)	> 8–16 (n = 610)	Farms (n = 267)	
*C. bovis*	113 (21.4)	114 (24.6)	129 (21.1)	154 (57.7)*	356 / 22.2*
*C. ryanae*	54 (10.2)	64 (13.8)	83 (13.6)	104 (38.9)*	201 / 12.5*
*C. parvum*	61 (11.6)	23 (4.9)	14 (2.3)	42(15.7) *	98 / 6.1*
*C. andersoni*	5 (0.9)	22 (4.7)	27 (4.4)	27 (10.1)	54 / 3.4
*C. bovis* and *C. ryanae*	4 (0.7)	3 (0.6)	3 (0.5)	7 (2.6)	10 / 0.6
*C. parvum* and *C. bovis*	2 (0.4)	0	0	2 (0.7)	2 / 0.1
*C. baileyi*	0	1 (0.2)	1 (0.2)	2 (0.7)	2 / 0.1
*C. suis*	0	0	2 (0.3)	2 (0.7)	2 / 0.1
Total	239 (45.3)	227 (48.9)	259 (42.4)	233 (87.2)	725 (45.3)

*Estimated values did not consider mixed *C. bovis* and *C. ryanae or C. parvum* and *C. bovis* infections.

However, statistically significant differences were solely observed between animals at the age of 4–8 and 8–16 weeks (χ^2^ = 4.19; *p* = 0.041). Six different species were detected being *C. bovis* the most common in the studied cattle population (χ^2^ = 189.13, *p* < 0.001) (Table 3).

Prevalence of *C. bovis* in the youngest animals, 49.8% (CI 95%: 43.3–56.3) was similar to that in those above 8 weeks of age, 50.9% (CI 95%: 44.7–57.2). This was also the case with *C. ryanae*, 24.2% (CI 95%: 18.9–30.2) and 33.2% (CI 95%: 27.4–39.3) , respectively. *C. bovis* and *C. ryanae* were the most common species in calves > 8 weeks of age. The overall prevalence of *C. parvum* in cattle from Poland up to 16 weeks of age was estimated at 6.2% (100/1601). In contrast to infections with *C. parvum,* the number of infections with *C. andersoni* increased with an animal age. *C. baileyi, C. suis* and mixed infections caused by the species pairs of *C. bovis* and *C. ryanae* or *C. parvum* and *C. bovis* were found in cattle only occasionally (Table 3).

### Geographical prevalence of *Cryptosporidium* species in cattle

There were also differences observed in the prevalence of infections caused by particular parasite species between provinces (Table 4). *C. bovis* predominated in LS, PM and ZP compared to PK (χ^2^ = 6.6–2.4; *p* = 0.01–0.028; OR 2.4–2.7), LD (χ^2^ = 11.0–0.13; *p* = 0.001–0.004; OR 3.2–2.8), and WM (χ^2^ = 5.8–3.8; *p* = 0.016–0.05; OR 2.2–1.9). Significant differences were also seen between SL and DS (χ^2^ = 5.1, *p* = 0.024; OR 0.4), OP and DS (χ^2^ = 6.1; *p* = 0.013; OR = 0.4), OP and SK (χ^2^ = 4.5; *p* = 0.033; OR = 0.5); PK and SK (χ^2^ = 7.9; *p* = 0.005; OR 0.3), PK and KP (χ^2^ = 4.3; *p* = 0.039; OR 0.4), LD and PL (χ^2^ = 3.9; *p* = 0.047; OR 0.5), LD and KP (χ^2^ = 7.6; *p* = 0.006; OR 0.4). Furthermore, *C. bovis* infections predominated in DS (χ^2^ = 15.5–4.3; *p* = 0.001–0.038; OR 4.0–1.9) when compared to other provinces except for SK and KP. Likewise, differences in *Cryptosporidium* prevalence were found between the following regions SK and LD (χ^2^ = 13.0, p ≤ 0.001; OR 3.5), MP (χ^2^ = 5.5; *p* = 0.019; OR 2.1), LB (χ^2^ = 4.7; *p* = 0.03; OR 2.0), MZ (χ^2^ = 4.7; *p* = 0.03; OR 2.0), and WM (χ^2^ = 7.2; *p* = 0.007; OR 2.4. Except for MP and WM, *C. ryanae* ranked first in PM than in other Polish provinces (χ^2^ = 26.2–5.7; *p* = 0.001–0.017; OR 0.05–0.2). It also prevailed in ZP, SL, OP, PK, SK compared to MP (χ^2^ = 7.0–3.9; *p* = 0.008–0.047; OR 3.5–2.5). Other significant differences in prevalence of infections caused by this parasite species were observed between LS and PM (χ^2^ = 8.7; *p* = 0.003; OR 7.2), LS and PL (χ^2^ = 6.8; *p* = 0.009; OR 0.4), ZP and PL (χ^2^ = 4.1; *p* = 0.043; OR 0.5), OP and WM (χ^2^ = 5.8; *p* = 0.016; OR 3.0), DS and PL (χ^2^ = 4.8; *p* = 0.028; OR 0.4), SK and WM (χ^2^ = 4.4; *p* = 0.037; OR 2.5), LD and PL (χ^2^ = 5.8; *p* = 0.016; OR 0.4), MP and LB (χ^2^ = 3.9; *p* = 0.047; OR 0.4), MP and PL (χ^2^ = 15.0; p ≤ 0.001; OR 0.2), MP and MZ (χ^2^ = 4.7; *p* = 0.03; OR 0.4), LB and PL (χ^2^ = 4.1, *p* = 0.043; OR 0.5), PL and KP (χ^2^ = 7.8; *p* = 0.005; OR 2.8), PL and WP (χ^2^ = 10.1; *p* = 0.001; OR 3.4), PL and WM (χ^2^ = 13.3; p ≤ 0.001; OR 4.4).

**Table 4:** Results on prevalence of *Cryptosporidium* species detected in cattle in each Polish province.

*Cryptosporidium* species	Prevalence by province (%)															
	LS (n = 108)	PM (n = 108)	ZP (n = 108)	SL (n = 72)	OP (n = 72)	DS (n = 90)	PK (n = 72)	SK (n = 108)	LD (n = 108)	MP (n = 108)	LB (n = 108)	PL (n = 108)	MZ (n = 108)	KP (n = 108)	WP (n = 107)	WM (n = 108)
*Cryptosporidium bovis*	28 (25.9)	32 (29.6)	31 (2.9)	13 (18.0)	12 (16.7)	31 (34.4)	10 (13.9)	35 (32.4)	12 (11.1)	20 (18.5)	21 (19.4)	23 (21.3)	21 (19.4)	29 (26.8)	20 (18.7)	18 (16.7)
*Cryptosporidium ryanae*	11 (10.2)	2 (1.8)	14 (12.9)	11 (15.3)	13 (18.0)	11 (12.2)	12 (16.7)	18 (16.7)	14 (12.9)	7 (8.5)	16 (14.8)	28 (26)	17 (15.7)	12 (11.1)	7 (6.5)	8 (7.4)
*Cryptosporidium parvum*	3 (2.8)	18 (16.7)	13 (12.0)	–	2 (2.8)	1 (1.1)	8 (11.1)	3 (2.8)	12 (11.1)	2 (1.8)	5 (4.6)	5 (4.6)	10 (9.2)	6 (5.5)	6 (5.6)	4 (3.7)
*Cryptosporidium andersoni*	–	2 (1.8)	–	7 (9.7)	7 (9.7)	4 (4.4)	1 (1.4)	1 (0.9)	4 (3.7)	1 (0.9)	2 (1.8)	9 (8.3)	–	4 (3.7)	6 (5.6)	6 (5.5)
*Cryptosporidium suis*	–	–	–	–	–	–	–	–	–	–	–	1 (0.9)	–	–	1 (0.9)	–
*Cryptosporidium baileyi*	1 (0.9)	–	–	1 (1.4)	–	–	–	–	–	–	–	–	–	–	–	–
*Cryptosporidium bovis* and *Cryptosporidium ryanae*	2 (1.8)	–	2 (1.8)	1 (1.4)	1 (1.4)	1 (1.1)	–	–	–	–	–	–	–	–	3 (2.8)	–
*Cryptosporidium parvum* and *Cryptosporidium bovis*	–	–	–	–	–	–	–	–	1 (0.92)	–	–	1 (0.9)	–	–	–	–

LS—Lubuskie, PM—Pomorskie, ZP—Zachodniopomorskie, SL—Śląskie, OP—Opolskie, DS—Dolnośląskie, PK—Podkarpackie, SK—Świętokrzyskie, LD—Łódzkie, MP—Małopolskie, LB—Lubelskie, PL—Podlaskie, MZ—Mazowieckie, KP—Kujawsko–Pomorskie, WP—Wielkopolskie, WM—Warmińsko–Mazurskie.

“– “ not detected.

*C. parvum* occurred with significantly higher prevalences in PM (χ^2^ = 14.1–6.6, *p* = 0.001–0.01; OR = 3.4–17.8) relative to the remaining provinces with the exception of ZP, PK, LD, and MZ. Likewise, its dominance was noted in ZP compared to SL, OP, DS, SK, MP, LB, and WM (χ^2^ = 9.3–3.9; *p* = 0.002–0.049; OR 2.8–12.2). Regional differences in the number of *C. parvum* isolations were found between LS, SL, OP, DS and PK (χ^2^ = 6.5–3.9; *p* = 0.011–0.049; OR 0.1–0.2) as well as between LS, SL, OP, DS, SK and LD (χ^2^ = 9.3–4.8; *p* = 0.002–0.028; OR 0.1–0.2). They were also found for LS and PM (χ^2^ = 11.9; *p* ≤ 0.001; OR 0.1), LS and ZP (χ^2^ = 6.7; *p* = 0.009; OR 0.2), PK and MP (χ^2^ = 5.4; *p* = 0.02; OR 6.6), LD and MP (χ^2^ = 8.7; *p* = 0.003; OR 7.2), LD and LB (χ^2^ = 3.9; *p* = 0.049; OR 2.8), LS and MZ (χ^2^ = 4.0; *p* = 0.045; OR 0.3), DS and MZ (χ^2^ = 6.2, *p* = 0.013; OR 0.1), SL and MZ (χ^2^ = 5.4, *p* = 0.02; OR value not estimated due to a low sample size), SK and MZ (χ^2^ = 4.0; *p* = 0.045; OR 0.3), MP and MZ (χ^2^ = 5.6; *p* = 0.017; OR 0.2), LD and WM (χ^2^ = 5.2; *p* = 0.023; OR 3.6), PK and SK (χ^2^ = 3.9; *p* = 0.049; OR 4.4).

The provinces at the opposite extreme were SL for *C. parvum* and LS, ZP and MZ for *C. andersoni*, where these parasites were not detected. Isolations of *C. suis* and *C. baileyi* from cattle were achieved in PL and WP and in LS and SL respectively (Table 4). Mixed infections of *C. bovis* and *C. ryanae* with a 2.8% frequency were observed in WP and below 1.8% in LS, ZP, SL, OP and DS.

## Breed–related prevalence of *Cryptosporidium* spp*.*

Besides BB and SL animals other breeds were found positive for *Cryptosporidium* DNA. The majority of infections (71.7%; 520/725) were detected in dairy cattle of HO breed (Table 5). *Cryptosporidium* prevalences were also high in MM (8.3%; 60/725) and SM (5.2%; 38/725) breeds. Analysing occurrence of infections within the breed, *C. parvum* prevailed in NCB (χ^2^ = 442; *p* < 0.001; OR 38.3), *C. ryanae* in LM (χ^2^ = 10.3; *p* = 0.01; OR value not estimated due to low sample size) and *C. andersoni* in MS cattle (χ^2^ = 103; *p* > 0.01; OR 4.6). It is noteworthy that also differences in prevalence of parasite species between some breeds were observed. For instance, *C. bovis* was significantly more often detected in HO compared to LM (χ^2^ = 3.8; *p* = 0.05). This relation was also seen between other breeds such as LM and SM (χ^2^ = 5.7; *p* < 0.05) as well as for LM and RW (χ^2^ = 4.3; *p* < 0.05). Likewise, *C. bovis* predominated in SM (χ^2^ = 5.2; *p* < 0.05) and RW (χ^2^ = 5.5; *p* < 0.05) cattle compared to animals of MS breed. *C. ryanae* infections prevailed respectively in HO (χ^2^ = 11.5; *p* < 0.01) and LM (χ^2^ = 12.8; *p* < 0.01) breeds relative to LM and SM cattle.

**Table 5:** Results on prevalence of detected *Cryptosporidium spp.* in cattle of different breeds in Poland.

*Cryptosporidium* species	Cattle breed																	
	HO (n = 1112)	LM (n = 72)	MM (n = 145)	SM (n = 93)	ZB (n = 22)	RW (n = 61)	MO (n = 10)	NCB (n = 28)	BS (n = 1)	AN (n = 6)	CH (n = 7)	BB (n = 1)	SL (n = 1)	RP (n = 7)	MS (n = 22)	JE (n = 1)	MDM (n = 12)	Total
*Cryptosporidium bovis*	245 (22.0)	23 (31.9)	32 (22.0)	15 (16.1)	4 (18.2)	10 (16.4)	2 (20.0)	6 (21.4)	1 (100)	2 (33.3)	2 (28.6)	–	–	5 (71.4)	9 (40.9)	–	–	356
*Cryptosporidium ryanae*	155 (13.9)	–	15 (10.3)	15 (16.1)	2 (18.2)	5 (8.2)	1 (10.0)	3 (10.7)	–	2 (33.3)	1 (14.3)	–	–	–	1 (4.5)	1 (100)	–	201
*Cryptosporidium parvum*	74 (6.6)	6 (8.3)	6 (4.1)	4 (4.3)	–	–	–	–	–	–	–	–	–	–	1 (4.5)	–	7 (58.3)	98
*Cryptosporidium andersoni*	36 (3.2)	2 (2.8)	5 (3.4)	4 (4.3)	–	5 (8.2)	–	1 (3.6)	–	–	–	–	–	–	1 (4.5)	–	–	54
*Cryptosporidium suis*	2 (0.2)	–	–	–	–	–	–	–	–	–	–	–	–	–	–	–	–	2
*Cryptosporidium baileyi*	–	1 (1.4)	–	–	–	–	–	1 (3.6)	–	–	–	–	–	–	–	–	–	2
*Cryptosporidium bovis and Cryptosporidium ryanae*	6 (0.5)	–	2 (1.8)	–	–	–	–	2 (7.1)	–	–	–	–	–	–	–	–	–	10
*Cryptosporidium parvum and Cryptosporidium bovis*	2 (0.2)	–	–	–	–	–	–	–	–	–	–	–	–	–	–	–	–	2
Total	520 (71.1)	32 (44.4)	60 (41.4)	38 (40.9)	6 (27.3)	20 (32.8)	3 (30.0)	13 (46.4)	1 (100)	4 (66.7)	3 (42.8)	–	–	5 (71.4)	12 (54.5)	1 (100)	7 (58.3)	725

HO—Polish Black and White Holstein*–*Friesian, LM—Limousine, MM—Mixed meat breeds, SM—Simmental, ZB—Polish Black and White, RW—Polish Red and White Holstein*–*Friesian, MO—Montbéliarde, NCB—Black and White lowland, BS—Brown Swiss, AN—Aberdeen Angus, CH—Charolaise, BB—Belgian Blue, SL– Salers, RP—Polish Red, MS—Mixed dairy breeds, JE—Jersey, MDM—Mixed dairy–meat breeds.

“– “ not detected.

## Discussion

Molecular methods have become irreplaceable in the detection and identification of *Cryptosporidium* species. PCR–based methods are gradually replacing microscopic methods in epidemiological studies on *Cryptosporidium* prevalence in livestock^23–29^ as they offer higher sensitivity and allow precise and direct identification of parasite species^30,31^. In this study, the prevalence of *Cryptosporidium* infections in cattle was only determined on the basis of molecular testing followed by the identification of parasite species using RFLP analysis. It was fast and reliable alternative to amplicon sequencing which often fails for samples containing a mixture of closely related parasite strains or species. To our knowledge this is the first nationwide epidemiological study on *Cryptosporidium* prevalence conducted in Europe, although a similar type of research has already been performed in China^31^. In contrast to our work, other *Cryptosporidium* studies carried out in cattle in Europe were only limited to animal populations kept regionally in each country^32–37^.

The prevalence of *Cryptosporidium* infections in cattle from Poland at the age of 1 to 16 weeks was 45.3%, with only significant difference in parasite prevalence between the older animal groups. Nevertheless, regional differences in prevalence of infections related to animals age were observed. In the north–western, central and eastern regions of Poland, a higher number of infections was found in the youngest animals up to 8 weeks of age. This can to some extend be explained by the presence of a higher cattle population housed in these regions with majority of animals represented by dairy breeds. There is a marked difference from the prevalence found in our previous study aiming at detection and molecular identification of *Cryptosporidium* species in cattle between the ages of 1 day and 6 years, in which it was on average 17%^9^. This large discrepancy could have resulted from testing of approximately threefold higher number of animals which gave more accurate data on *Cryptosporidium* prevalence at province level as well as from different sampling plan used in the current study. The narrow age range (1–16 weeks) of the sampled cattle may have also contributed to the results obtained as in older animals which were not tested in this study, *Cryptosporidium* infections occur less frequently^38,39^. It should be emphasised that sampled animals originated from randomly selected farms of different sizes which were located in the districts of their provinces with the highest cattle populations. Therefore, it is likely that higher density of animal population in the studied regions could have facilitated infection spread between animals and farms. This observation has previously been shown on commercial cattle farms^40–42^. Likewise, an association between dairy herd size and an increased risk of *Cryptosporidium* infection has been demonstrated^43^. Also shown in this study, the intensive cattle rearing system was mainly associated with dairy farming, which might have influence on prevalence of *Cryptosporidium* infection, as in all provinces with infection rate above 50%, dairy farming was the main rearing system. A direct comparison of the results obtained in this study with those of other European studies is difficult, as most of them focused on small animal populations and a discrete region^25,42^ or were limited to selected farms^44^. Certainly, the higher *Cryptosporidium* prevalence of the present study cannot be attributed to differences in sample processing and detection methodology, because those studies also employed molecular methods. As an outcome comparable to our results, a high 48.6% rate of *Cryptosporidium* prevalence was observed in cattle in a cohort study conducted in Sweden^45^ and 43.8% prevalence in dairy cattle herds in Cyprus^46^. Contrastingly to the European data, a low 14.1% infection rate was recorded in a large population study on pre–weaned cattle under the age of 3 months from northwest China^47^. Surprisingly, a low 9.9% infection prevalence was estimated for diarrhoeic calves in Korea^48^. Differences in infection prevalence can be found despite age similarity of the tested animals and size similarity of the sampled animal population. Various factors such as geographical location, environmental conditions, animal breed, husbandry system and rearing conditions may have an influence on the results obtained.

In the current study the majority of infections in calves up to 4 months were caused by *C. bovis* and *C*. *ryanae*. The species distribution in animals has changed in Poland over time^9^ as currently *C. parvum* infections were reported less frequently. A similar infection pattern of detected species with dominating *C. bovis* and *C*. *ryanae* has also been shown in several studies from different parts of the world^45,49^. As demonstrated here, *C. parvum* was not the dominant species in healthy calves up to 8 weeks of age, and this finding is in agreement with previous observations^45^. Consequently, it is not surprising that different distribution patterns of C. *parvum* have been found depending on the health status of the tested animals^33,50,51^. In this study, the overall prevalence of *C. parvum* in cattle in Poland was estimated at 6.2%. It was also found that animals under the age of 1 month were not the major host for *C. parvum* , and the number of *C. parvum–*positive samples decreased with a higher animal age. These results are consistent with our previous observations on *C. parvum* prevalence in cattle in Poland^9^. Among the *Cryptosporidium* species identified in this study, *C. parvum* is considered the most important zoonotic species.

However, in this study unusual *Cryptosporidium* species for a bovine host, namely *C. baileyi* and *C. suis*, were detected in the herds investigated, in which infected animals were carrying those *Cryptosporidium* species asymptomatically. The calves positive for *C. baileyi* and *C. suis* represented different breeds (LM, HO and NCB) as well as rearing types (meat, dairy and dairy–meat varieties). Thus far, *C. baileyi*^52^, *C. suis*^53,54^, *Cryptosporidium scrofarum*^55^, *Cryptosporidium xiaoi*^34^, *Cryptosporidium serpentis*, *Cryptosporidium tyzzeri*^56^, *C. felis*^57^ and *Cryptosporidium occultus* (previously known as *C. suis*–like)^34,55^ have been detected in grazing ruminants without evidence that they were causing disease. In this study, the presence of *C. baileyi* and *C. suis* in cattle might be associated with an accidental oocyst ingestion with contaminated feed. By examining the routes whereby *C. baileyi* could have been transmitted, it seems likely that feed contamination occurred as a result of environmental parasite abundance linked to extensive poultry farming in areas where the calves were being reared. However, if this finding would be reported as repeatable, a further investigation would be required to reveal any possible parasite–host interactions. In the present study, mixed infections of *C. bovis* and *C. ryanae* or *C. parvum* and *C. bovis* were detected. They were previously found sporadically in young cattle in Poland^9^, China (*C. parvum* and *C. bovis*)^29^ and Brazil (*C. parvum* and *C. bovis*; *C. parvum* and *C. ryanae*; *C. parvum* and *C. andersoni*)^39^. Majewska et al.^58^ also detected concurrent parasite infections in Polish cattle, but in contrast to our findings, they were caused by *C. parvum* and *C. andersoni.*

Significant differences in *Cryptosporidium* prevalence (from 27.8% to 62%) in Polish provinces were observed. Those results are also in agreement with reports from Korea describing a wide range of prevalence rates of infections between regions^48^. In this study a higher prevalence of *Cryptosporidium* infections in particular provinces was not always correlated with a higher population of housed animals, for example, in MP province with the lowest 27.8% *Cryptosporidium* prevalence, the cattle population was similar to this observed in PM with 50% frequency of infections. Of note is that the prevalence above 50% was mostly observed in provinces in which the tested animals were housed on farms with an intensive cattle rearing system which is mainly associated with dairy farming. Likewise, there were differences observed between species distribution and the region of the country. There were also no differences found in the frequency of infections caused by particular *Cryptosporidium* species in animals representing the intensive cattle rearing system compared to general cattle population.

Little is known about *Cryptosporidium* occurrence in cattle of different breeds. When data on *Cryptosporidium* prevalence was broken down by breed type, a relationship between the infecting specific parasite species and the animal breed was found. For instance, *C. parvum* prevailed in NCB, *C. ryanae* in LM and *C. andersoni* in MS cattle. However, the observed in this study prevalence of breed–associated parasite species should be interpreted with caution because of differences in the numbers of tested animals of each production purpose: the herds were of mostly dairy breeds with HO dominating animals and meat breeds were the next largest proportion. In this light, the current results do not necessarily indicate any greater breed sensitivity to *Cryptosporidium* infection. It seems to be justified that a higher number of animals of a particular breed type be tested to reveal exact parasite–host interactions. However, breed–related prevalence of *Cryptosporidium* infections has also been demonstrated in a comparison between European–bred animals, Zebu and animals cross–bred with Zebu^39^. While this relation was not indicated in our previous study related to *Cryptosporidium* epidemiology in cattle, it was evident for goats which showed breed–related differences in parasite prevalence^59^. In this study the distribution of *Cryptosporidium* species at the farm level was not analysed. Considering the randomness of sampling and varied ages of calves sampled on each farm, those farm–level variables would not guarantee obtaining reliable species distribution results. As a final observation of a possible limitation, although our sampling scheme covered all Polish provinces, the number of sampled animals may not reflect the actual population size in each region. Nevertheless, the sampling plan used allowed to estimate the prevalence of *Cryptosporidium* infections with 95% probability for the results with a binomial distribution. Only prevalences assessed for particular Polish provinces can be characterized by lower probability. However, the large, total sample size allowed to draw valid conclusions. Furthermore, testing of young animals < 4 months of age in which *Cryptosporidium* infections are mostly prevalent can be considered a sentinel study for occurrence of *Cryptosporidium* species in the population.

## Conclusions

The results of this cross–sectional population study provide evidence that in clinically healthy population of young calves up to 4 months from Poland, *C. bovis* and *C. ryanae* infections predominate relegating *C. parvum* to third in prevalence. Mixed infections caused by *C. bovis* and *C. ryanae* as well as *C. parvum* and *C. bovis* were also observed. Likewise, detection of the unusual *C. baileyi* and *C. suis* in grazing animals was achieved. This is the first report describing their presence in the population of cattle in Poland.

## Data Availability

The sequences of *18S rRNA* fragment gene of *C. baileyi* and *C. suis* strains were deposited into the NCBI GenBank under accession numbers OP090504-OP090507.

## Abbreviations

*18S** rRNA*: Small subunit rRNA gene
AN: Aberdeen Angus
BB: Belgian Blue
BS: Brown Swiss
*C. andersoni*: *Cryptosporidium andersoni*
*C. baileyi*: *Cryptosporidium baileyi*
*C. bovis*: *Cryptosporidium bovis*
*C. parvum*: *Cryptosporidium parvum*
*C. ryanae*: *Cryptosporidium ryanae*
*C. suis*: *Cryptosporidium suis*
CH: Charolaise
DS: Dolnośląskie (province)
HO: Polish Black and White Holstein–Friesian
JE: Jersey
KP: Kujawsko-Pomorskie (province)
LB: Lubelskie (province)
LD: Łódzkie (province)
LM: Limousine
LS: Lubuskie (province)
MDM: Mixed dairy-meat breeds
MM: Mixed meat breeds
MO: Montbéliarde
MP: Małopolskie (province)
MS: Mixed dairy breeds
MZ: Mazowieckie (province)
NCB: Black and White lowland
OP: Opolskie (province)
PCR: Polymerase chain reaction
PK: Podkarpackie (province)
PL: Podlaskie (province)
PM: Pomorskie (province)
RFLP: Restriction fragment length polymorphism
RP: Polish Red
RW: Polish Red and White Holstein–Friesian
SL: Salers
SL: Śląskie (province)
SM: Simmental
SK: Świętokrzyskie (province)
WM: Warmińsko-Mazurskie (province)
WP: Wielkopolskie (province)
ZB: Polish Black and White
ZP: Zachodniopomorskie (province)
